# Supplementary material for: Neurologic Deficit Score at 4–5 Days Post-eCPR Predicts Long-Term Brain Dysfunction in Rats Following Cardiac Arrest
Source: Biomolecules. 2025 May 16;15(5):732. doi: 10.3390/biom15050732 (PMC12110119; doi:10.3390/biom15050732)
Supplement: Supplementary file 1 [file biomolecules-15-00732-s001.zip › biomolecules-3505378-supplementary.pdf]

Table S1. Antibodies used for immunohistochemistry

Antibody	Dilution	Antigen retrieval	Secondary antibody	Chromogen
OGDH Polyclonal antibody<br>Proteintech #15212-1-AP	1:800	pH9<br>TRIS EDTA Buffer	Bright Vision Goat anti Rabbit<br>HRP RTU (KL DPVR 110 HRP)	Bright DAB<br>(WellMed BV, DAB Bright BS04-110)
HMOX2 Polyclonal antibody<br>Proteintech #14817-1-AP	1:600	pH6<br>Citrate Buffer	Bright Vision Goat anti Rabbit<br>HRP RTU (KL DPVR 110 HRP)	Bright DAB<br>(WellMed BV, DAB Bright BS04-110)
Iba1 Polyclonal antibody<br>Wako #019-19741	1:30000	pH6<br>Citrate Buffer	Bright Vision Goat anti Rabbit<br>HRP RTU (KL DPVR 110 HRP)	DAB Quanto<br>(Thermo Scientific #TA-125-QHDX)
GFAP Polyclonal antibody<br>Dako #Z0334	1:5000	Pronase	Bright Vision Goat anti Rabbit<br>HRP RTU (KL DPVR 110 HRP)	DAB Quanto<br>(Thermo Scientific #TA-125-QHDX)

Table S2. Primers used for gene expression analysis.

Accesion number	Target	Primer sequence	Position on + strand	Product length (bp)	exon junction in	Intron size (bp)	Source
NM_012583.2	HPRT	CTC ATG GAC TGA TTA TGG ACA GGA C<br>GCA GGT CAG CAA AGA ACT TAT AGC C	179<br>301	123	fwd. primer	-	(1)
NM_017101.1	Cyc	TAT CTG CAC TGC CAA GAC TGA GTG<br>CTT CTT GCT GGT CTT GCC ATT CC	381<br>507	127	product	199	(1)
NM_012580.2	HO-1	CCA GCC ACA CAG CAC TAC<br>GCG GTC TTA GCC TCT TCT G	441<br>733	293	-	-	(1)
NM_024387.2	HO-2	GGG GAA GGG ACC CAG TTC TA<br>CCA GGG TAC CTT TGT CTG GC	725<br>1016	292	rev. primer<br>product	-<br>677	(2)
NM_013091.1	TNFR1	GTG CCA CAA AGG AAC CTA CTT G<br>AGG CTG GAG TTA GGG GCT TA	420<br>1048	629	fwd. primer<br>product	-<br>3290	(3)
NM_001017461.1	OGDH (E1)	CAT GGG TCC CGA GCA TTC CT<br>GCA GCA AGA TCT GTC GTC GC	2433<br>2623	191	fwd. primer<br>product	-<br>540	(3)
NM_199385.2	DLD (E3)	CTG TGT GGA AGG AAT GGC GG<br>TGA TAA GGT CGG ATG TGC ATG G	1199<br>1556	358	rev. primer<br>product	-<br>4277	(3)

References

1. Postl A, Zifko C, Hartl RT, Ebel T, Miller I, Moldzio R, et al. Transient increase of free iron in rat livers following hemorrhagic-traumatic shock and reperfusion is independent of heme oxygenase 1 upregulation. Shock. 2011;36(5):501-9.
2. Warenits AM, Hatami J, Mullebner A, Ettl F, Teubenbacher U, Magnet IAM, et al. Motor Cortex and Hippocampus Display Decreased Heme Oxygenase Activity 2 Weeks After Ventricular Fibrillation Cardiac Arrest in Rats. Front Med (Lausanne). 2020;7:513.
3. Mkrtchyan GV, Ucal M, Mullebner A, Dumitrescu S, Kames M, Moldzio R, et al. Thiamine preserves mitochondrial function in a rat model of traumatic brain injury, preventing inactivation of the 2-oxoglutarate dehydrogenase complex. Biochim Biophys Acta Bioenerg. 2018;1859(9):925-31.

Supplementary Figure

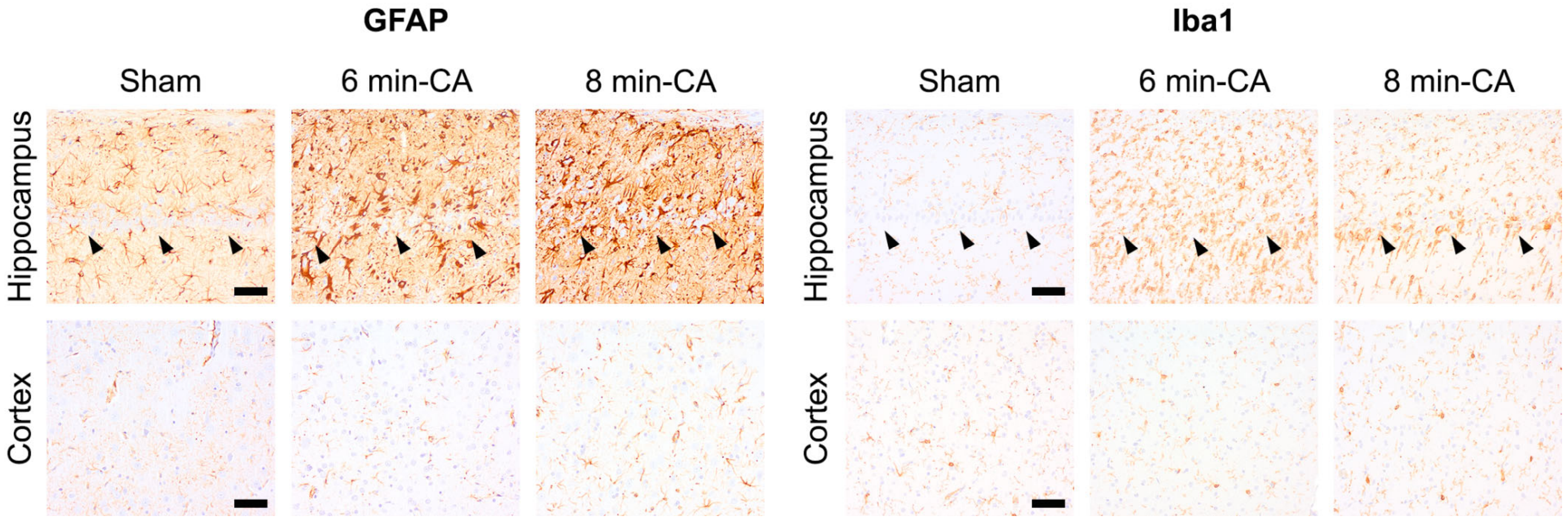

Fig. S1: Representative images of the glial reaction in Hc and mC tissue of rats 14 days after CA. *Immunohistochemistry was performed with antibodies against glial fibrillary acidic protein (GFAP) for detection of astrocytes (left panel) and ionized calcium binding adaptor molecule 1 (Iba1) for detection of microglia (right panel). The Hc shows increased signal intensity of both, astrocytes and microglia, after 6 and 8 min of CA compared to sham. The pyramidal layer of the hippocampal CA1 region is depicted by arrowheads (top). In contrast, the mC does not show any glial reaction after 6 and 8 min of CA compared to sham (bottom). Bars = 20µm.*

Accesion number	Target	Primer sequence	Position on + strand	Product length (bp)	exon junction in	Intron size (bp)	Source
NM_012583.2	HPRT	CTC ATG GAC TGA TTA TGG ACA GGA C	179	123	fwd. primer	-	(1)
		GCA GGT CAG CAA AGA ACT TAT AGC C	301				
NM_017101.1	Cyc	TAT CTG CAC TGC CAA GAC TGA GTG	381	127	product	199	(1)
		CTT CTT GCT GGT CTT GCC ATT CC	507				
NM_012580.2	HO-1	CCA GCC ACA CAG CAC TAC	441	293	-	-	(1)
		GCG GTC TTA GCC TCT TCT G	733				
NM_024387.2	HO-2	GGG GAA GGG ACC CAG TTC TA	725	292	rev. primer<br>product	-<br>677	(2)
		CCA GGG TAC CTT TGT CTG GC	1016				
NM_013091.1	TNFR1	GTG CCA CAA AGG AAC CTA CTT G	420	629	fwd. primer<br>product	-<br>3290	(3)
		AGG CTG GAG TTA GGG GCT TA	1048				
NM_001017461.1	OGDHC E1k	CAT GGG TCC CGA GCA TTC CT	2433	191	fwd. primer<br>product	-<br>540	(3)
		GCA GCA AGA TCT GTC GTC GC	2623				
NM_199385.2	OGDHC E3	CTG TGT GGA AGG AAT GGC GG	1199	358	rev. primer<br>product	-<br>4277	(3)
		TGA TAA GGT CGG ATG TGC ATG G	1556				
